# *catena*-Poly[bis­[(1,10-phenanthroline)iron(II)]-bis­(μ-5-carboxy­benzene-1,3-dicarboxyl­ato)]

**DOI:** 10.1107/S160053680804018X

**Published:** 2008-12-06

**Authors:** Lin Cheng, Ya-Wen Zhang, Yan-Yan Sun, Jian-Quan Wang

**Affiliations:** a School of Chemistry and Chemical Engineering, Southeast University, Nanjing 211189, People’s Republic of China

## Abstract

The asymmetric unit of the title compound, [Fe(C_9_H_4_O_6_)(C_12_H_8_N_2_)(H_2_O)]_*n*_, contains one Fe^II^ cation, one 5-carboxy­benzene-1,3-dicarboxyl­ate dianion (Hbtc), one 1,10-phenanthroline (phen) ligand and one water mol­ecule. The Fe^II^ centre displays a distorted octa­hedral geometry, being surrounded by one phen ligand, two μ_2_-O atoms of two carboxyl­ate groups from two Hbtc ligands, one O atom from one carboxyl­ate of another Hbtc ligand and one terminal water mol­ecule. One carboxyl­ate group ligates two Fe^II^ cations in a μ_1,1_ mode, while the other carboxyl­ate groups bonds to only one Fe atom. The crystal structure is stabilized by O—H⋯O hydrogen bonds.

## Related literature

For related structures, see: Plater *et al.* (2001 ▶). For general background, see: Yang *et al.* (2008 ▶); Bradshaw *et al.* (2004 ▶); Chui *et al.* (1999 ▶).
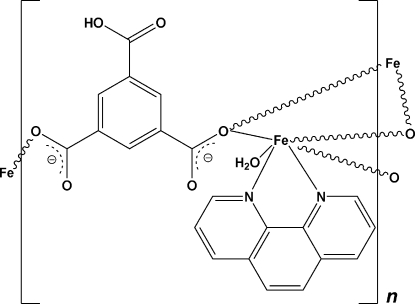


## Experimental

### 

#### Crystal data

[Fe(C_9_H_4_O_6_)(C_12_H_8_N_2_)(H_2_O)]
*M* *_r_* = 462.19
Triclinic, 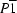

*a* = 9.5925 (15) Å
*b* = 10.8971 (16) Å
*c* = 11.1998 (17) Å
α = 96.221 (3)°
β = 111.320 (2)°
γ = 111.736 (2)°
*V* = 972.3 (3) Å^3^
*Z* = 2
Mo *K*α radiation
μ = 0.82 mm^−1^
*T* = 293 (2) K
0.25 × 0.22 × 0.18 mm

#### Data collection

Bruker APEX CCD diffractometer
Absorption correction: multi-scan (*SADABS*; Sheldrick, 2000 ▶) *T*_min_ = 0.821, *T*_max_ = 0.866
7689 measured reflections
3798 independent reflections
3228 reflections with *I* > 2σ(*I*)
*R*_int_ = 0.024

#### Refinement

*R*[*F* ^2^ > 2σ(*F* ^2^)] = 0.041
*wR*(*F* ^2^) = 0.096
*S* = 1.05
3798 reflections
292 parameters
H atoms treated by a mixture of independent and constrained refinement
Δρ_max_ = 0.37 e Å^−3^
Δρ_min_ = −0.22 e Å^−3^

### 

Data collection: *SMART* (Bruker, 2000 ▶); cell refinement: *SAINT* (Bruker, 2000 ▶); data reduction: *SAINT*; program(s) used to solve structure: *SHELXTL* (Sheldrick, 2008 ▶); program(s) used to refine structure: *SHELXTL*; molecular graphics: *SHELXTL*; software used to prepare material for publication: *SHELXTL*.

## Supplementary Material

Crystal structure: contains datablocks I, global. DOI: 10.1107/S160053680804018X/bt2819sup1.cif

Structure factors: contains datablocks I. DOI: 10.1107/S160053680804018X/bt2819Isup2.hkl

Additional supplementary materials:  crystallographic information; 3D view; checkCIF report

## Figures and Tables

**Table 1:** Hydrogen-bond geometry (Å, °)

*D*—H⋯*A*	*D*—H	H⋯*A*	*D*⋯*A*	*D*—H⋯*A*
O1*W*—H1*WA*⋯O1^i^	0.85 (4)	1.79 (4)	2.602 (3)	159 (3)
O1*W*—H1*WB*⋯O4^ii^	0.80 (4)	1.94 (4)	2.740 (3)	172 (3)
O3—H3*A*⋯O6^iii^	0.79 (4)	1.86 (4)	2.622 (3)	160 (4)

Symmetry codes: (i) 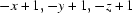; (ii) 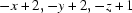; (iii) 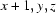.

**Fig. 1.**
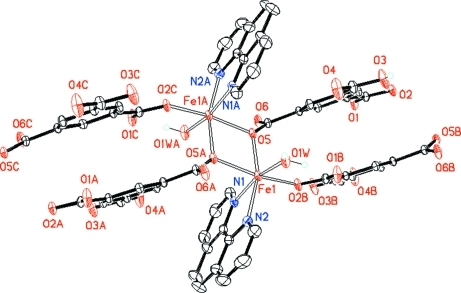
The Fe2 structure of the title compound with 30% thermal ellipsoids. All the H atoms on the C atoms are omitted for clarity. Symmetry codes: a: 1 - x, 2 - y, 1 - z; b: 1 - x, 1 - y, 1 - z; c: x, 1 + y, z.

**Fig. 2.**
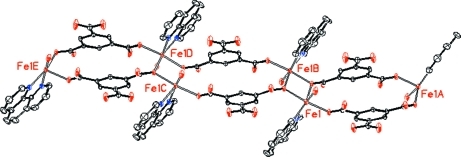
The one-dimensional chain of the title compound. All the H atoms have been omitted for clarity.

## supplementary crystallographic information

### Comment

Recent years have witnessed an explosion of great interest in hybrid organic–inorganic framework solids not only for their intriguing architectures and topologies, but also for their potential applications in optical, electrical, magnetic and microporous materials (Yang *et al.*, 2008). And benzene-1,3,5-tricarboxylic acid has been widely used to construct many novel and interesting metal–organic frameworks (Bradshaw *et al.*, 2004; Chui *et al.*, 1999). Herein, we present the synthesis and structural characterization of a new one-dimensional compound [Fe(Hbtc)(phen)]*n* (H*3*btc = benzene-1,3,5-tricarboxylic acid; phen = 1,10-phenanthroline) using H*3*btc as a ligand.

The asymmetric unit of the title compound, contains a Fe^II^ cation, a dianion of Hbtc and a chelating phen. In the compound, each Fe^II^ displays a distorted octahedral geometry, being surrounded by one phen ligand, two µ^2^-O atoms of two carboxylates coming from two Hbtc ligands, one O atom from one carboxylate of another Hbtc and one terminal water molecule. The Hbtc ligand coming from the deprotonation of two carboxylates of H*3*btc acts as a dianion, in which one carboxylate ligates two Fe^II^ cations in the µ_1,1_ mode with the Fe—O—Fe angle at 102.75 (7)° and Fe···Fe distance of 3.38 (3) Å, to form a Fe_2_ unit; while the other carboxylate adopts a monodentate mode. Thus, the adjacent Fe_2_ units are linked each other by a pair of tridentate Hbtc ligands in head-to-tail into a one-dimensional chain. The shortest Fe···Fe distance separated by Hbtc ligands is 10.90 (3) Å. Further, the one-dimensional chains are stabled by intrachain hydrogen bonding between coordinated water molecules and adjacent uncoordinated O atoms of monodentate carboxylates with the O···O distance of 2.602 (3) Å. Finally, the one-dimensional chains are further linked together by the interchain hydrogen bonding between uncoordinated carboxylates and uncoordinated O atoms of coordinated µ^2^-carboxylates as well as coordinated water molecules into a two-dimensional supramolecular network.

### Experimental

A mixture of H_3_btc (0.0210 g, 0.1 mmol), phen (0.0180 g, 0.1 mmol), FeCl_2_.4H_2_O (0.0199 g, 0.1 mmol) and H_2_O (8 ml) was heated in a 15 ml Teflon-lined autoclave at 160 ° for 3 d, followed by slow cooling (5 ° h^-1^) to room temperature. The resulting mixture was washed with water, and red block crystals were collected and dried in air (yield 32%, 14.8 mg) based on Fe^II^].

### Refinement

The H atoms bonded to O atom were located in a difference map and freely refined. Other H atoms were positioned geometrically and refined using a riding model with C—H = 0.93 Å and with *U*_iso_(H) = 1.2U_eq_(C).

### Crystal data

**Table:**

[Fe(C_9_H_4_O_6_)(C_12_H_8_N_2_)(H_2_O)]	*Z* = 2
*M**_r_* = 462.19	*F*(000) = 472
Triclinic, *P*1	*D*_x_ = 1.579 Mg m^−^^3^
Hall symbol: -P 1	Mo *K*α radiation, λ = 0.71073 Å
*a* = 9.5925 (15) Å	Cell parameters from 785 reflections
*b* = 10.8971 (16) Å	θ = 2.5–28.0°
*c* = 11.1998 (17) Å	µ = 0.82 mm^−^^1^
α = 96.221 (3)°	*T* = 293 K
β = 111.320 (2)°	Block, red
γ = 111.736 (2)°	0.25 × 0.22 × 0.18 mm
*V* = 972.3 (3) Å^3^	

### Data collection

**Table:**

Bruker APEX CCD diffractometer	3798 independent reflections
Radiation source: fine-focus sealed tube	3228 reflections with *I* > 2σ(*I*)
graphite	*R*_int_ = 0.024
φ and ω scans	θ_max_ = 26.0°, θ_min_ = 2.0°
Absorption correction: multi-scan (*SADABS*; Sheldrick, 2000)	*h* = −11→11
*T*_min_ = 0.821, *T*_max_ = 0.866	*k* = −13→13
7689 measured reflections	*l* = −13→13

### Refinement

**Table:**

Refinement on *F*^2^	Primary atom site location: structure-invariant direct methods
Least-squares matrix: full	Secondary atom site location: difference Fourier map
*R*[*F*^2^ > 2σ(*F*^2^)] = 0.041	Hydrogen site location: inferred from neighbouring sites
*wR*(*F*^2^) = 0.096	H atoms treated by a mixture of independent and constrained refinement
*S* = 1.05	*w* = 1/[σ^2^(*F*_o_^2^) + (0.0467*P*)^2^ + 0.209*P*] where *P* = (*F*_o_^2^ + 2*F*_c_^2^)/3
3798 reflections	(Δ/σ)_max_ < 0.001
292 parameters	Δρ_max_ = 0.37 e Å^−^^3^
0 restraints	Δρ_min_ = −0.21 e Å^−^^3^

### Special details

**Table:**

Geometry. All e.s.d.'s (except the e.s.d. in the dihedral angle between two l.s. planes) are estimated using the full covariance matrix. The cell e.s.d.'s are taken into account individually in the estimation of e.s.d.'s in distances, angles and torsion angles; correlations between e.s.d.'s in cell parameters are only used when they are defined by crystal symmetry. An approximate (isotropic) treatment of cell e.s.d.'s is used for estimating e.s.d.'s involving l.s. planes.
Refinement. Refinement of *F*^2^ against ALL reflections. The weighted *R*-factor *wR* and goodness of fit *S* are based on *F*^2^, conventional *R*-factors *R* are based on *F*, with *F* set to zero for negative *F*^2^. The threshold expression of *F*^2^ > σ(*F*^2^) is used only for calculating *R*-factors(gt) *etc*. and is not relevant to the choice of reflections for refinement. *R*-factors based on *F*^2^ are statistically about twice as large as those based on *F*, and *R*- factors based on ALL data will be even larger.

### Fractional atomic coordinates and isotropic or equivalent isotropic displacement parameters (Å^2^)

**Table:**

	*x*	*y*	*z*	*U*_iso_*/*U*_eq_	
Fe1	0.61551 (4)	0.98060 (3)	0.64542 (3)	0.02290 (12)	
C1	0.4072 (3)	0.4212 (2)	0.3242 (2)	0.0203 (5)	
C2	0.5643 (3)	0.4812 (2)	0.3283 (2)	0.0238 (5)	
H2A	0.6162	0.4266	0.3174	0.029*	
C3	0.6454 (3)	0.6223 (2)	0.3487 (2)	0.0240 (5)	
C4	0.5696 (3)	0.7048 (2)	0.3685 (2)	0.0239 (5)	
H4A	0.6239	0.7994	0.3829	0.029*	
C5	0.4132 (3)	0.6459 (2)	0.3669 (2)	0.0202 (5)	
C6	0.3329 (3)	0.5041 (2)	0.3441 (2)	0.0218 (5)	
H6A	0.2277	0.4643	0.3421	0.026*	
C7	0.3235 (3)	0.2700 (2)	0.3103 (2)	0.0244 (5)	
C8	0.8159 (3)	0.6886 (3)	0.3565 (3)	0.0320 (6)	
C9	0.3293 (3)	0.7313 (2)	0.3927 (2)	0.0234 (5)	
C10	0.2781 (3)	0.8910 (3)	0.6674 (3)	0.0393 (7)	
H10A	0.2341	0.8235	0.5880	0.047*	
C11	0.1720 (4)	0.8963 (4)	0.7242 (4)	0.0556 (9)	
H11A	0.0602	0.8326	0.6841	0.067*	
C12	0.2347 (5)	0.9959 (4)	0.8387 (4)	0.0585 (10)	
H12A	0.1653	1.0011	0.8773	0.070*	
C13	0.4026 (4)	1.0903 (3)	0.8989 (3)	0.0491 (8)	
C14	0.4804 (6)	1.1991 (5)	1.0193 (4)	0.0739 (12)	
H14A	0.4165	1.2100	1.0615	0.089*	
C15	0.6433 (6)	1.2854 (4)	1.0726 (4)	0.0753 (12)	
H15A	0.6891	1.3571	1.1492	0.090*	
C16	0.7479 (5)	1.2701 (3)	1.0150 (3)	0.0525 (9)	
C17	0.9206 (5)	1.3534 (4)	1.0703 (3)	0.0640 (10)	
H17A	0.9726	1.4269	1.1465	0.077*	
C18	1.0113 (4)	1.3257 (4)	1.0116 (3)	0.0648 (10)	
H18A	1.1262	1.3788	1.0487	0.078*	
C19	0.9317 (4)	1.2178 (3)	0.8961 (3)	0.0461 (7)	
H19A	0.9955	1.2009	0.8568	0.055*	
C20	0.6769 (4)	1.1637 (3)	0.8977 (3)	0.0349 (6)	
C21	0.5017 (4)	1.0749 (3)	0.8375 (3)	0.0340 (6)	
N1	0.4379 (3)	0.9776 (2)	0.7216 (2)	0.0291 (5)	
N2	0.7682 (3)	1.1375 (2)	0.8391 (2)	0.0322 (5)	
O1	0.1824 (2)	0.22146 (18)	0.3066 (2)	0.0449 (5)	
O2	0.4051 (2)	0.20291 (16)	0.30592 (18)	0.0295 (4)	
O3	0.8788 (3)	0.6020 (2)	0.3450 (3)	0.0548 (7)	
O4	0.8901 (2)	0.8094 (2)	0.3733 (3)	0.0625 (7)	
O5	0.42061 (19)	0.86215 (15)	0.44513 (16)	0.0249 (4)	
O6	0.1789 (2)	0.67369 (18)	0.3605 (2)	0.0378 (5)	
O1W	0.8228 (2)	0.9974 (2)	0.6184 (2)	0.0413 (5)	
H1WA	0.847 (4)	0.935 (4)	0.648 (4)	0.073 (12)*	
H1WB	0.902 (4)	1.053 (4)	0.613 (3)	0.065 (12)*	
H3A	0.971 (5)	0.642 (4)	0.353 (4)	0.093 (15)*	

### Atomic displacement parameters (Å^2^)

**Table:**

	*U*^11^	*U*^22^	*U*^33^	*U*^12^	*U*^13^	*U*^23^
Fe1	0.0206 (2)	0.01554 (19)	0.0350 (2)	0.00822 (15)	0.01468 (15)	0.00608 (14)
C1	0.0202 (12)	0.0166 (12)	0.0260 (12)	0.0084 (10)	0.0118 (10)	0.0063 (9)
C2	0.0235 (12)	0.0199 (12)	0.0328 (13)	0.0127 (10)	0.0146 (10)	0.0058 (10)
C3	0.0207 (12)	0.0199 (12)	0.0334 (13)	0.0083 (10)	0.0148 (10)	0.0066 (10)
C4	0.0213 (12)	0.0156 (12)	0.0339 (13)	0.0064 (10)	0.0134 (11)	0.0051 (10)
C5	0.0178 (11)	0.0171 (12)	0.0282 (12)	0.0081 (10)	0.0124 (10)	0.0053 (10)
C6	0.0154 (11)	0.0202 (12)	0.0289 (12)	0.0065 (10)	0.0106 (10)	0.0056 (10)
C7	0.0224 (13)	0.0180 (12)	0.0333 (13)	0.0087 (10)	0.0128 (11)	0.0080 (10)
C8	0.0254 (13)	0.0264 (14)	0.0492 (16)	0.0112 (12)	0.0220 (12)	0.0100 (12)
C9	0.0238 (13)	0.0179 (12)	0.0319 (13)	0.0106 (10)	0.0142 (11)	0.0074 (10)
C10	0.0308 (15)	0.0423 (17)	0.0510 (17)	0.0155 (13)	0.0242 (14)	0.0153 (14)
C11	0.0382 (18)	0.077 (3)	0.073 (2)	0.0316 (18)	0.0369 (18)	0.034 (2)
C12	0.068 (2)	0.090 (3)	0.070 (2)	0.057 (2)	0.056 (2)	0.043 (2)
C13	0.068 (2)	0.062 (2)	0.0497 (19)	0.0456 (19)	0.0408 (18)	0.0234 (17)
C14	0.109 (4)	0.094 (3)	0.062 (2)	0.068 (3)	0.057 (3)	0.021 (2)
C15	0.115 (4)	0.077 (3)	0.049 (2)	0.059 (3)	0.039 (2)	0.001 (2)
C16	0.077 (2)	0.0453 (19)	0.0344 (16)	0.0313 (19)	0.0193 (17)	0.0080 (14)
C17	0.081 (3)	0.042 (2)	0.0379 (18)	0.018 (2)	0.0070 (18)	−0.0034 (15)
C18	0.051 (2)	0.049 (2)	0.049 (2)	0.0016 (17)	−0.0024 (17)	0.0040 (17)
C19	0.0371 (17)	0.0394 (17)	0.0456 (17)	0.0084 (14)	0.0109 (14)	0.0088 (14)
C20	0.0495 (17)	0.0289 (14)	0.0306 (14)	0.0212 (13)	0.0174 (13)	0.0106 (12)
C21	0.0480 (17)	0.0347 (15)	0.0366 (15)	0.0275 (14)	0.0247 (13)	0.0178 (13)
N1	0.0299 (12)	0.0272 (12)	0.0390 (12)	0.0157 (10)	0.0204 (10)	0.0121 (10)
N2	0.0311 (12)	0.0250 (12)	0.0351 (12)	0.0091 (10)	0.0123 (10)	0.0078 (10)
O1	0.0269 (10)	0.0238 (10)	0.0952 (16)	0.0128 (9)	0.0344 (11)	0.0246 (11)
O2	0.0312 (10)	0.0181 (9)	0.0514 (11)	0.0141 (8)	0.0265 (9)	0.0132 (8)
O3	0.0302 (12)	0.0282 (11)	0.116 (2)	0.0128 (10)	0.0452 (13)	0.0108 (12)
O4	0.0372 (12)	0.0277 (12)	0.141 (2)	0.0130 (10)	0.0576 (14)	0.0292 (13)
O5	0.0248 (9)	0.0146 (8)	0.0364 (9)	0.0090 (7)	0.0146 (7)	0.0055 (7)
O6	0.0203 (9)	0.0232 (9)	0.0718 (13)	0.0096 (8)	0.0239 (9)	0.0070 (9)
O1W	0.0315 (11)	0.0269 (11)	0.0819 (16)	0.0154 (10)	0.0366 (11)	0.0248 (11)

### Geometric parameters (Å, °)

**Table:**

Fe1—O1W	2.063 (2)	C11—C12	1.356 (5)
Fe1—O2^i^	2.0873 (16)	C11—H11A	0.9300
Fe1—O5^ii^	2.1554 (15)	C12—C13	1.393 (5)
Fe1—N1	2.157 (2)	C12—H12A	0.9300
Fe1—O5	2.1746 (17)	C13—C21	1.404 (4)
Fe1—N2	2.192 (2)	C13—C14	1.431 (5)
C1—C2	1.384 (3)	C14—C15	1.339 (5)
C1—C6	1.387 (3)	C14—H14A	0.9300
C1—C7	1.502 (3)	C15—C16	1.423 (5)
C2—C3	1.390 (3)	C15—H15A	0.9300
C2—H2A	0.9300	C16—C20	1.402 (4)
C3—C4	1.395 (3)	C16—C17	1.404 (5)
C3—C8	1.487 (3)	C17—C18	1.358 (5)
C4—C5	1.388 (3)	C17—H17A	0.9300
C4—H4A	0.9300	C18—C19	1.388 (4)
C5—C6	1.392 (3)	C18—H18A	0.9300
C5—C9	1.504 (3)	C19—N2	1.329 (3)
C6—H6A	0.9300	C19—H19A	0.9300
C7—O1	1.240 (3)	C20—N2	1.358 (3)
C7—O2	1.262 (3)	C20—C21	1.430 (4)
C8—O4	1.196 (3)	C21—N1	1.352 (3)
C8—O3	1.312 (3)	O2—Fe1^i^	2.0873 (16)
C9—O6	1.228 (3)	O3—H3A	0.79 (4)
C9—O5	1.290 (3)	O5—Fe1^ii^	2.1554 (15)
C10—N1	1.318 (3)	O1W—H1WA	0.85 (4)
C10—C11	1.396 (4)	O1W—H1WB	0.80 (4)
C10—H10A	0.9300		
			
O1W—Fe1—O2^i^	89.06 (7)	C12—C11—H11A	120.5
O1W—Fe1—O5^ii^	96.92 (7)	C10—C11—H11A	120.5
O2^i^—Fe1—O5^ii^	166.10 (7)	C11—C12—C13	120.3 (3)
O1W—Fe1—N1	166.83 (9)	C11—C12—H12A	119.9
O2^i^—Fe1—N1	87.61 (7)	C13—C12—H12A	119.9
O5^ii^—Fe1—N1	89.21 (7)	C12—C13—C21	117.1 (3)
O1W—Fe1—O5	100.00 (8)	C12—C13—C14	124.5 (3)
O2^i^—Fe1—O5	89.38 (6)	C21—C13—C14	118.4 (3)
O5^ii^—Fe1—O5	77.25 (6)	C15—C14—C13	121.4 (3)
N1—Fe1—O5	92.70 (7)	C15—C14—H14A	119.3
O1W—Fe1—N2	92.32 (9)	C13—C14—H14A	119.3
O2^i^—Fe1—N2	103.69 (7)	C14—C15—C16	121.6 (3)
O5^ii^—Fe1—N2	88.65 (7)	C14—C15—H15A	119.2
N1—Fe1—N2	76.12 (8)	C16—C15—H15A	119.2
O5—Fe1—N2	162.22 (7)	C20—C16—C17	117.3 (3)
C2—C1—C6	119.2 (2)	C20—C16—C15	118.8 (3)
C2—C1—C7	120.7 (2)	C17—C16—C15	123.9 (3)
C6—C1—C7	119.9 (2)	C18—C17—C16	119.4 (3)
C1—C2—C3	120.6 (2)	C18—C17—H17A	120.3
C1—C2—H2A	119.7	C16—C17—H17A	120.3
C3—C2—H2A	119.7	C17—C18—C19	119.7 (3)
C2—C3—C4	119.7 (2)	C17—C18—H18A	120.1
C2—C3—C8	121.2 (2)	C19—C18—H18A	120.1
C4—C3—C8	119.0 (2)	N2—C19—C18	122.9 (3)
C5—C4—C3	120.1 (2)	N2—C19—H19A	118.5
C5—C4—H4A	120.0	C18—C19—H19A	118.5
C3—C4—H4A	120.0	N2—C20—C16	122.8 (3)
C4—C5—C6	119.3 (2)	N2—C20—C21	117.4 (2)
C4—C5—C9	122.0 (2)	C16—C20—C21	119.8 (3)
C6—C5—C9	118.73 (19)	N1—C21—C13	122.4 (3)
C1—C6—C5	121.0 (2)	N1—C21—C20	117.5 (2)
C1—C6—H6A	119.5	C13—C21—C20	120.1 (3)
C5—C6—H6A	119.5	C10—N1—C21	118.4 (2)
O1—C7—O2	125.2 (2)	C10—N1—Fe1	126.62 (19)
O1—C7—C1	118.0 (2)	C21—N1—Fe1	114.93 (17)
O2—C7—C1	116.7 (2)	C19—N2—C20	117.7 (2)
O4—C8—O3	122.9 (2)	C19—N2—Fe1	128.4 (2)
O4—C8—C3	123.5 (2)	C20—N2—Fe1	113.40 (17)
O3—C8—C3	113.5 (2)	C7—O2—Fe1^i^	129.65 (15)
O6—C9—O5	124.0 (2)	C8—O3—H3A	110 (3)
O6—C9—C5	118.6 (2)	C9—O5—Fe1^ii^	125.52 (15)
O5—C9—C5	117.3 (2)	C9—O5—Fe1	131.19 (15)
N1—C10—C11	122.7 (3)	Fe1^ii^—O5—Fe1	102.75 (6)
N1—C10—H10A	118.6	Fe1—O1W—H1WA	106 (2)
C11—C10—H10A	118.6	Fe1—O1W—H1WB	141 (2)
C12—C11—C10	118.9 (3)	H1WA—O1W—H1WB	109 (3)

Symmetry codes: (i) −*x*+1, −*y*+1, −*z*+1; (ii) −*x*+1, −*y*+2, −*z*+1.

### Hydrogen-bond geometry (Å, °)

**Table:**

*D*—H···*A*	*D*—H	H···*A*	*D*···*A*	*D*—H···*A*
O1W—H1WA···O1^i^	0.85 (4)	1.79 (4)	2.602 (3)	159 (3)
O1W—H1WB···O4^iii^	0.80 (4)	1.94 (4)	2.740 (3)	172 (3)
O3—H3A···O6^iv^	0.79 (4)	1.86 (4)	2.622 (3)	160 (4)

Symmetry codes: (i) −*x*+1, −*y*+1, −*z*+1; (iii) −*x*+2, −*y*+2, −*z*+1; (iv) *x*+1, *y*, *z*.
